# Inferior vena cava tumor thrombus that directly infiltrated from paracaval lymph node metastases in a patient with recurrent hepatocellular carcinoma

**DOI:** 10.1186/1477-7819-11-177

**Published:** 2013-08-06

**Authors:** Shinya Imada, Kohei Ishiyama, Kentaro Ide, Tsuyoshi Kobayashi, Hironobu Amano, Hirotaka Tashiro, Koji Arihiro, Hiroshi Aikata, Kazuaki Chayama, Hideki Ohdan

**Affiliations:** 1 Gastroenterological and Transplant Surgery, Applied Life Science, Institute of Biomedical & Health Sciences, Hiroshima University, 1-2-3 Kasumi, Minami-ku, Hiroshima 734-8551, Japan; 2 Department of Pathology, Hiroshima University Hospital, Hiroshima, Japan; 3 Gastroenterology and Metabolism, Applied Life Sciences, Institute of Biomedical & Health Sciences, Hiroshima University, Hiroshima, Japan

**Keywords:** Hepatocellular carcinoma, Lymph node metastases, Inferior vena cava tumor thrombus

## Abstract

Herein, we present the case of a patient with recurrent hepatocellular carcinoma (HCC) who had paracaval lymph node (LN) metastases with an inferior vena cava (IVC) tumor thrombus after a hepatectomy. A 65-year-old man with chronic hepatitis B virus infection received an extended anterior segmentectomy because of two hepatic tumors, located in segments 7 and 8. Histological examination of both resected specimens showed mostly moderately differentiated HCC with some poorly differentiated areas, and liver cirrhosis (A2/F4). Because the patient had an elevated α-fetoprotein serum level, abdominal computed tomography (CT) was performed. Abdominal CT revealed a 9-mm-diameter recurrent tumor in hepatic segment 3 and paracaval LN metastases with an IVC tumor thrombus at 8 months after the first operation. The patient received transcatheter arterial chemoembolization as treatment for the intrahepatic recurrence, following resection of the paracaval LN metastases and removal of the IVC tumor thrombus. In this case, the paracaval LN metastases had directly infiltrated the IVC via the lumbar veins, resulting in an IVC tumor thrombus, which usually develops from an intrahepatic tumor via the hepatic vein. The development of an IVC tumor thrombus with HCC recurrence, as in this case, is very rare, and based on a PubMed search, we believe this report may be the first to describe this condition.

## Background

Hepatocellular carcinoma (HCC) is a highly malignant form of cancer, which recurs frequently after hepatectomy [1]. The most common recurrent sites are the residual liver and lung; however, occurrence of lymph node (LN) metastases after hepatectomy is unusual [2]. Some studies have reported that metastatic LNs from HCC tend not to spread to the surrounding tissue [3]. Furthermore, in only 0.53% of patients with HCC did the inferior vena cava (IVC) have tumor invasion [4] that directly progressed from intrahepatic HCC. Therefore, local resection of HCC is a curative treatment to improve patient survival. In our case, metastases from the LNs on the paracaval site, which is an uncommon site for HCC metastases, directly infiltrated into the IVC and developed into an IVC tumor thrombus. Although some investigators have reported that metastatic LNs infiltrated the portal venous (PV) wall and developed into PV tumor thrombi in patients with HCC [3], no reports have described IVC tumor thrombi from LN metastases in patients with recurrent HCC. In this report, we describe a rare progressive pattern of a recurrent HCC with LN metastases, which showed an infiltrative growth pattern.

## Case presentation

**Figure 1:**
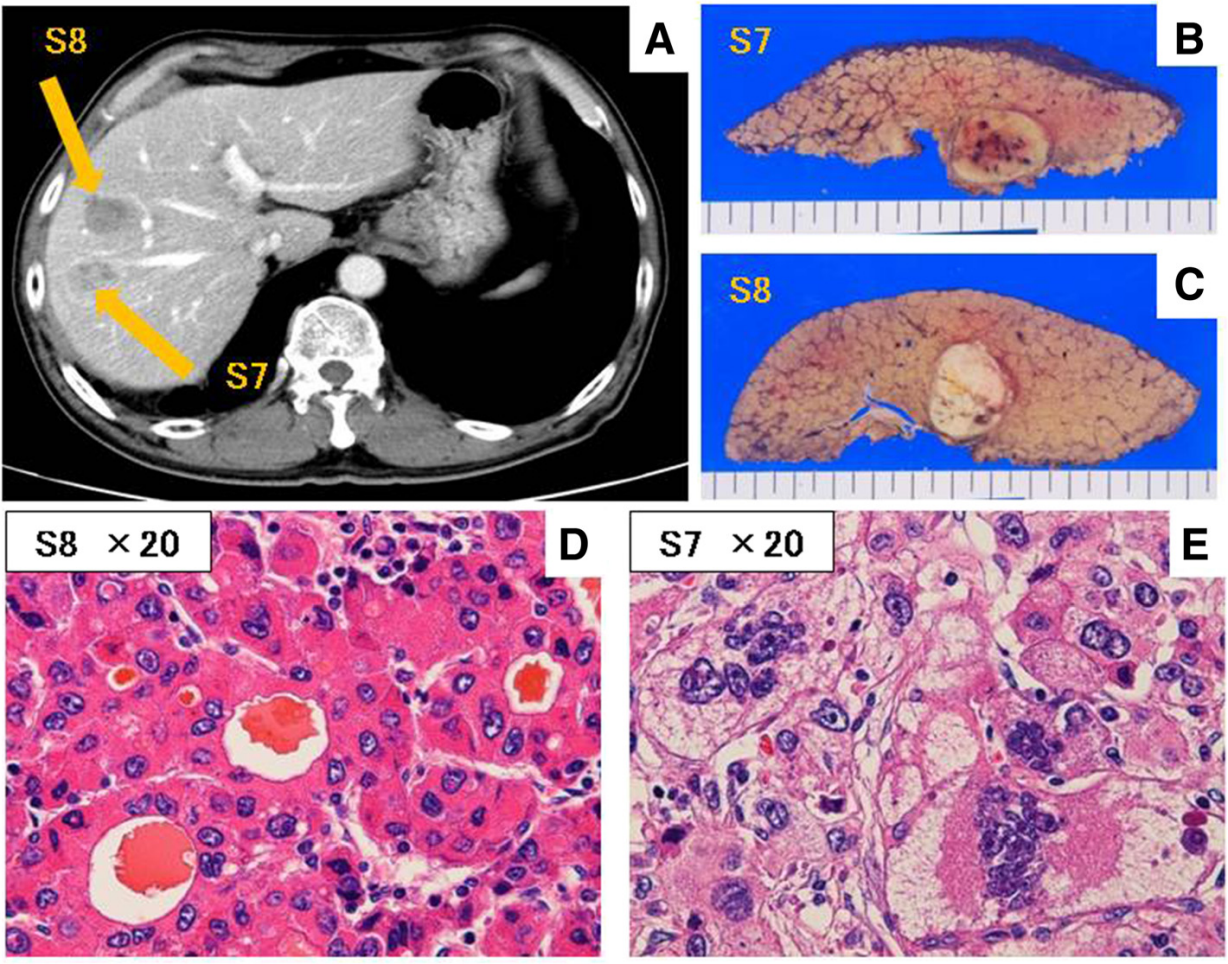
**The primary hepatocellular carcinoma. (A)** Dynamic computed tomography revealed a 20-mm-diameter hypervascular mass in segment 7 and a 20-mm-diameter hypovascular mass in segment 8. **(B, C)** Gross appearance of the two resected hepatic tumors. **(D, E)** Microscopic examination of the two specimens revealed moderately differentiated HCC. A few of the HCC cells from segment 7 were poorly differentiated, and there was evidence of tumor invasion into the portal vein (H&E, ×20).

**Figure 2:**
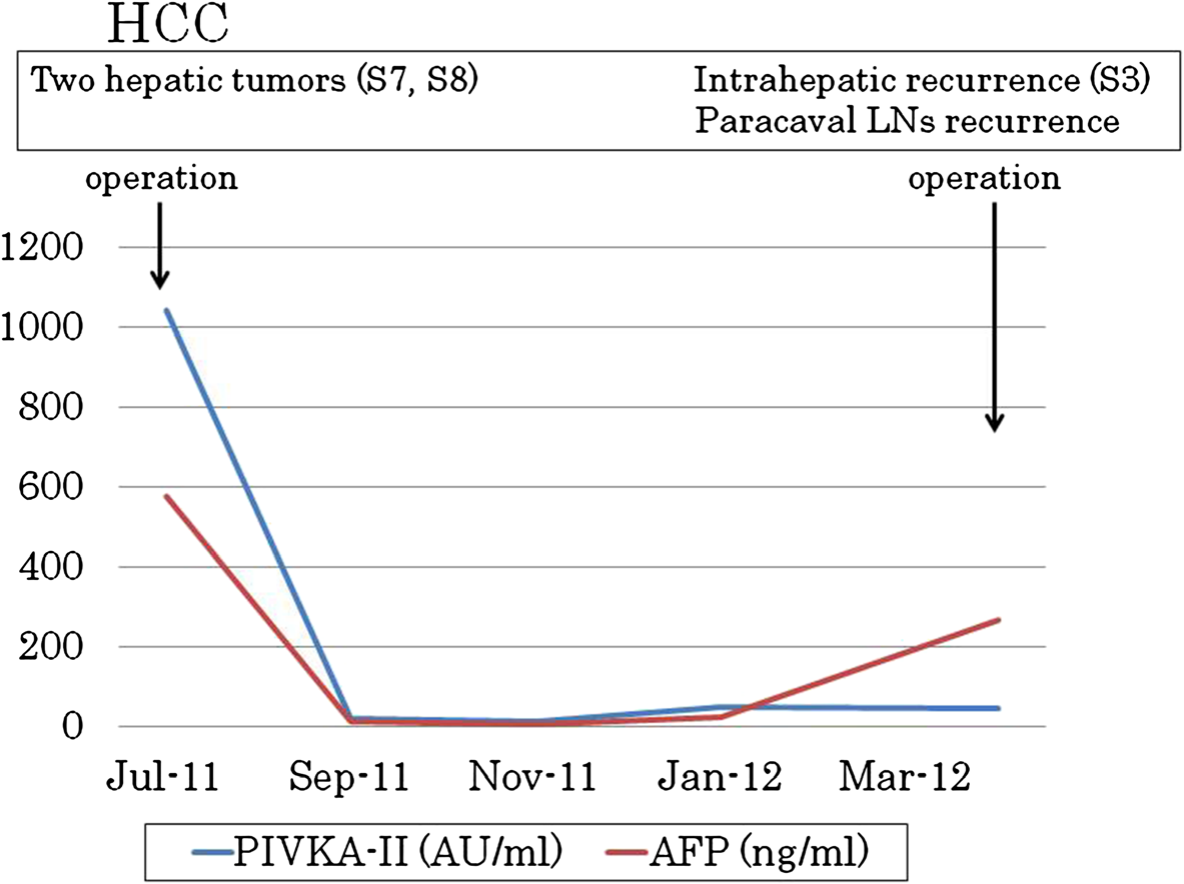
Clinical course of the tumor markers.

**Figure 3:**
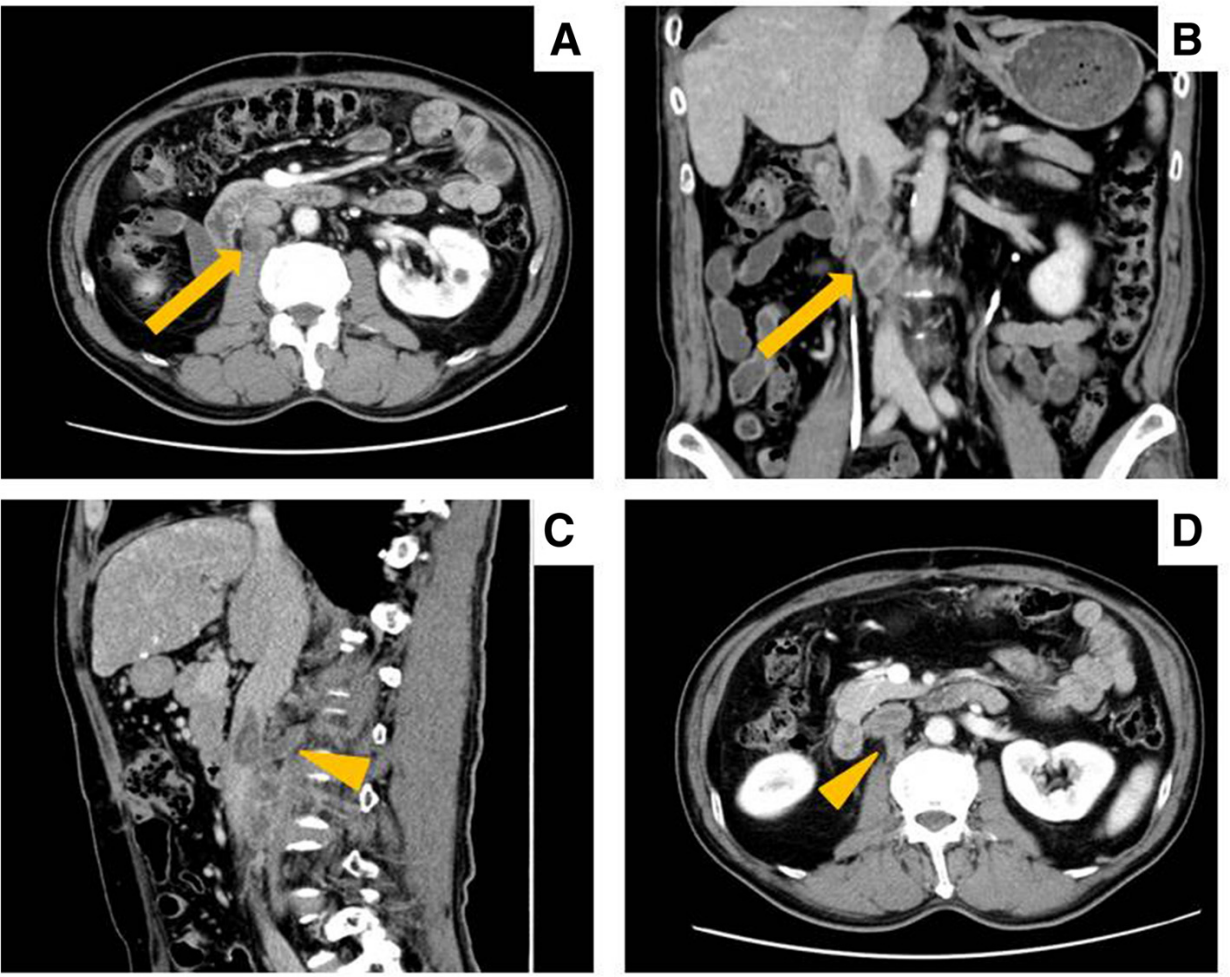
**The paracaval lymph node metastases with an inferior vena cava tumor torombus. (A, B)** Computed tomography revealed both enlarged lymph nodes (diameter, 3 cm; *arrow*), which were located behind the IVC, and **(C, D)** IVC tumor thrombus, which directly infiltrated through the lumbar vein (*arrowhead*).

**Figure 4:**
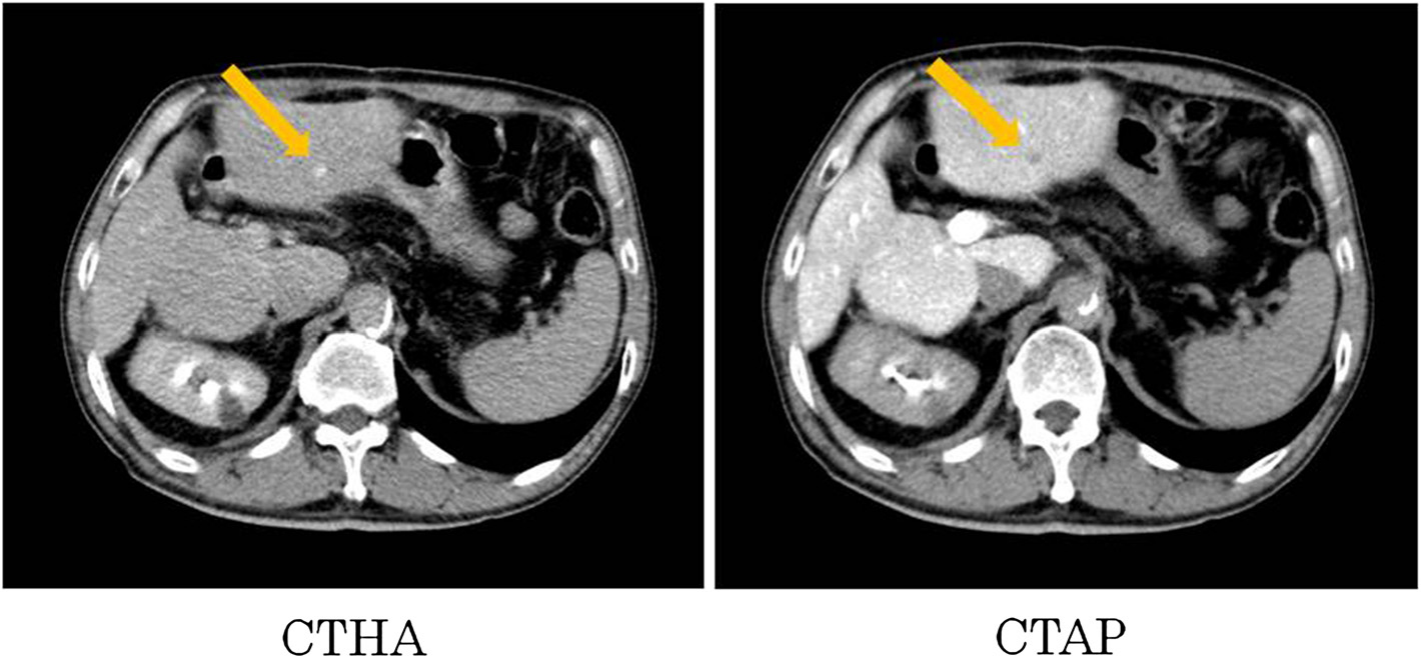
Computed tomography angiography revealed a 9-mm-diameter intrahepatic recurrence in segment 3.

A 65-year-old man with hepatitis B virus (HBV)-related cirrhosis was admitted to our institution for the treatment of recurrent intrahepatic HCC and paracaval LN metastases, with an IVC invasion and an IVC tumor thrombus. Eight months before this visit, he had undergone a curative operation for HCC at our institution. This operation involved an extended anterior segmentectomy, including resection of hepatic segment 7, for the treatment of two hepatic tumors, and the histological findings of both resected specimens showed mostly moderately differentiated HCC, with some poorly differentiated areas, and liver cirrhosis (LC; A2/F4; Figure 1). During physical examination, there was no tenderness in the abdomen, and no mass was palpable at the time of the administration. The patient’s blood biochemical values showed elevated levels of both serum α-fetoprotein (AFP; 266 ng/ml; normal, <6.5 ng/ml) and protein induced by vitamin K absence or antagonist II (PIVKA-II; 46 mAU/ml; normal, <40 mAU/ml) (Figure 2). Computed tomography (CT) revealed both enlarged LNs, measuring 3 cm in diameter, located behind the IVC and development of an IVC tumor thrombus apart from liver (Figure 3). Also, preoperative images showed the continuity of the intravascular lumen and the formation of thrombus consecutively from enlarged LNs through lumbar vein. Additionally, CT angiography revealed a 9-mm-diameter intrahepatic tumor in segment 3; this tumor had high attenuation on CT during arteriography (CTA) and low attenuation on CT during arterioportography (CTAP; Figure 4). Positron-emission tomography (PET) with ^18^ F-fluorodeoxyglucose (^18^ F-FDG) revealed no other areas of increased uptake, except for an intense area of increased uptake (SUVmax, 4.0) in the region that corresponded to the enlarged LNs, as previously seen with CT imaging. On the basis of these findings, we preoperatively diagnosed this condition as recurrence of intrahepatic carcinoma and paracaval LN metastases, with IVC invasion and thrombus formation. For treating the intrahepatic recurrence, the patient received transcatheter arterial chemoembolization (TACE) at the time of the CT angiography. Finally, as there was no metastasis in any other organs and the HCC was well controlled, we decided to perform surgical treatment for the LN metastases as a curative treatment.

**Figure 5:**
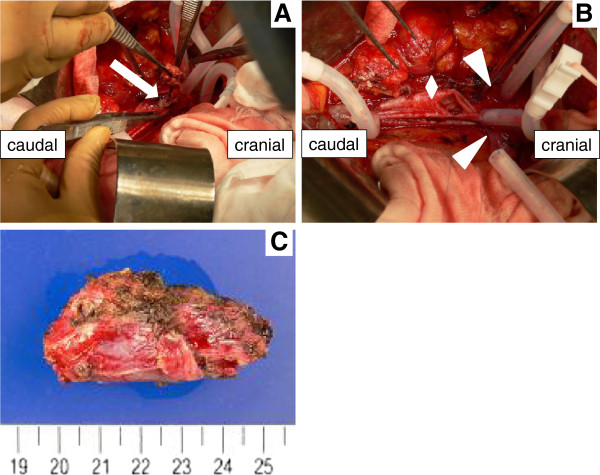
**The intraoperative findings and resected specimens. A, B**: Incision of the anterior part of the IVC (diamond) and thrombectomy of the IVC (arrow) after complete exposure and taping of both the renal veins (arrowhead) and IVC. **C**: Gross appearance of the resected LNs.

**Figure 6:**
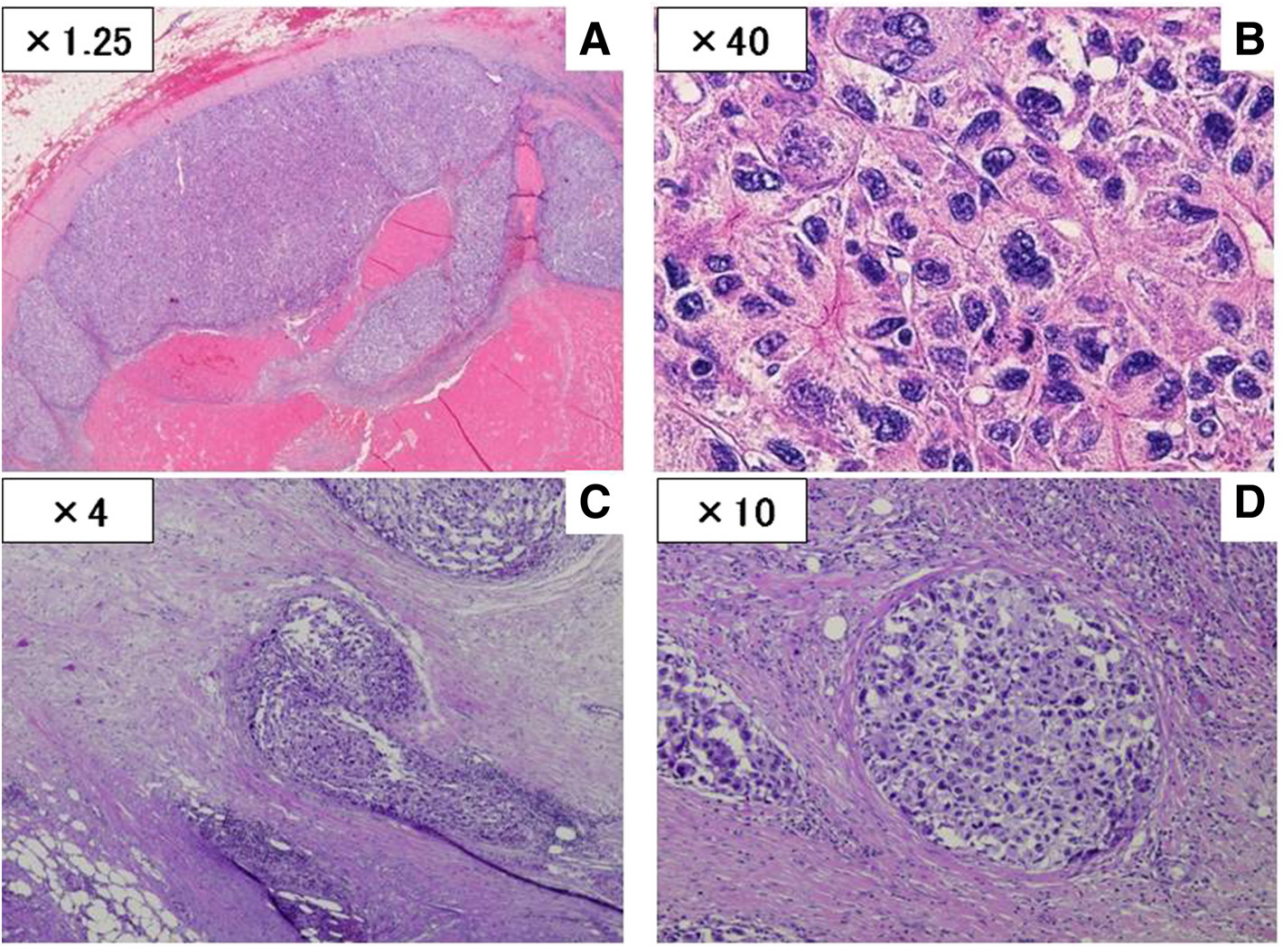
**The pathological examination of the resected LNs and IVC thrombus. A, B**: The pathological findings from the resected LNs revealed giant atypical and pleomorphic HCC cells (H&E; **A**, ×1.25; **B**, ×40). **C, D**: the HCC cells infiltrated into the drainage vein of the lymph nodes (H&E; **C**, ×4; **D**, ×10).

During the surgical procedure, we made an incision on the anterior aspect of the IVC and performed a thrombectomy for the IVC tumor thrombus, after clamping both renal veins and the IVC segment, because the cranial side of the IVC tumor thrombus extended under the bifurcation of the renal vein. Subsequently, we performed thrombectomy for the tumor from the lumbar vein, followed by dissection of the lumbar vein (Figure 5). The tumor growth was spreading from the lumbar vein, which branched from the IVC. Additionally, the intraoperative finding of this case revealed that the border between the IVC thrombus and the wall of the blood vessel was clear just like in the preoperative images. Therefore, we performed a lymphadenectomy after closing the IVC with a continuous suture. LNs located in a mass behind the IVC adhered to the peripheral tissue, but we could complete the surgical procedure with no macroscopic residual tumor. Pathological examination of the resected LNs and IVC thrombus revealed giant atypical and pleomorphic HCC cells and not vascularized tissue, consistent with a diagnosis of HCC (Figure 6A, B). In addition, the HCC cells located in the lymphatic vessel invaded the drainage vein of resected LNs (Figure 6C, D). Finally, we diagnosed that LN metastases but not IVC wall metastases from HCC directly infiltrated into the IVC and developed into an IVC tumor thrombus.

Hematogenous metastasis from HCC is a well-known pathway of tumor infiltration and growth, and the most frequent extrahepatic metastatic sites are the lung, followed by the adrenal gland and the skeleton [5]. However, the incidence of an extrahepatic metastasis in HCC is extremely low [6]. Although autopsy studies have clarified that the frequency of LN metastases from HCC are 23.5–43.9%, the detection of LN metastases during any type of treatment is uncommon and accounts for only 1.0% of hepatectomy cases [2]. The hepatic lymphatic system has been well elucidated [7]. In most of the lymph nodes, lymph flows toward the hepatic hilum and runs into the intra-abdominal lymphatic system through the hepatoduodenal ligament. Therefore, almost all metastatic LNs from HCC are located in the hepatoduodenal ligament and on the posterior surface of the pancreas head [8]. However, the progression of liver cirrhosis leads to lymphatic obstruction and the formation of collateral routes for lymphatic drainage [2]. This phenomenon may produce skip metastases, which means no LN metastases may occur in the proximal hepatoduodenal ligament but rather at distant sites [9]. In our case, the metastatic LNs were located behind the IVC, and there were no LN metastases in the corresponding proximal hepatoduodenal ligament. During histological examination, the primary surgical specimen appeared as LC, and the formation of collateral routes for lymphatic drainage might have led to skip metastases.

Ueda et al. suggested that the main drainage routes of HCC lesions are the portal venules; therefore, HCC may have a tendency to infiltrate the portal vein and may easily create a PV tumor thrombus [10]. Morimoto et al. [3] reported a case of HCC with a PV tumor thrombus from posterior pancreaticoduodenal LN metastases. In general, the main drainage of metastatic LN lesions as well as primary HCC may be via the portal venules; therefore, if there are any metastases to LNs, a PV tumor thrombus may also be present. However, in our case, there was a consecutive tumor thrombus from the lumbar vein to the IVC. We assumed that the development of a tumor thrombus from the lumbar vein to the IVC was due to the presence of both lymphogenous and hematogenous metastases at the local site. This phenomenon greatly differs from the general findings, showing that paracaval LN metastases from solid tumors are generally hematogenous; however, this patient had bone and lung metastases after the second operation via hematogenous metastasis from the metastatic LNs. In previous studies that have reported resection of metastatic LNs from HCC, the growth of the metastatic focus was almost noninfiltrative, with no involvement of blood vessels [11]. In contrast, other investigators have reported that the pathological characteristics of metastatic LNs comprised those of poorly differentiated HCC and that the growth patterns of metastatic LNs were infiltrative in patients with PV tumor thrombus that developed from metastatic LNs. Corresponding with the findings of the latter report, a microscopic examination of the resected LN metastases in our case showed poorly differentiated HCC, with proliferation in a trabecular or solid pattern. Lee et al. [12] investigated the clinicopathological characteristics of HCC with LN metastases, and they concluded that having both an infiltrating type and a larger (>5 cm) tumor with microvascular invasion, as well as a high-grade histology were associated with higher incidences of LN metastases. Although the size of the HCC tumor during the first hepatectomy was less than 5 cm in the present case, histological examination showed that the HCC cells were giant atypical cells, including pleomorphic cells, and had spread to the surrounding normal liver tissue with no well-defined border and had invaded the portal vein. Our patient had chronic HBV infection, but Lee et al. [12] concluded that neither HBV infection nor hepatitis C virus infection was related to the development of LN metastases.

Livraghi et al. reported a cohort study of radiofrequency ablation (RFA) and demonstrated that complete ablation of lesions smaller than 2 cm is possible in more than 90% of cases, with a local recurrence rate of less than 1% [13]. Also, Bujold et al. conducted prospective trials of stereotactic body radiotherapy (SBRT) for advanced HCC and reported that SBRT could lead to sustained local control, associated with survival rates higher than historical controls, with a low risk of serious toxicity [14], although HCC with no metastasis, which is less than 5 cm in diameter, is candidate for SBRT in our country. It seemed that our patient was a candidate for RFA but not for SBRT based on the tumor size and location, but we performed TACE concurrently at the time of CT angiography except for RFA because of the start of additional prompt treatment for LN recurrence by controlling the intrahepatic lesion. Also, we selected aggressive oncological treatment as a part of multidisciplinary treatments for recurrence instead of conservative treatment. Currently, there is no consensus regarding treatment strategies for LN metastases in HCC patients [15]. Zeng et al. and Toya et al. retrospectively evaluated the role of radiotherapy (RT) for HCC patients with LN metastases [16,17]. They showed that the median survival time (MST) was 9.4 months for the RT group and 3.3 months for the non-RT group (*p* < 0.001), and they concluded that LN metastasis from HCC was sensitive to RT. Also, Hou et al. evaluated the influence of PV vs. IVC tumor thrombosis sites on the effectiveness of RT in advanced HCC with macrovascular invasion. They concluded that HCC patients with IVC thrombus treated with RT had a better response rate and longer survival than those with PV thrombus [18]. In contrast, Kobayashi et al. compared the treatment results of surgery and RT for LN metastasis from HCC. They showed that the MST of patients with single and multiple LN metastases after surgery was 52 and 14 month, respectively (*p* < 0.01) and concluded that selective lymphadenectomy of LN metastasis was a safe and efficacious procedure [11], although a previous report on regional lymphadenectomy demonstrated a high rate of liver failure [19]. After examining RT for treatment of LN metastasis, we selected a surgical treatment because of the high risk of pulmonary embolism due to the large size of IVC tumor thrombus with relatively rapid growth without metastasis in any other organs and the composition of a mass of LNs. Concerning chemotherapy, although Llovet et al. reported that sorafenib prolonged median survival time and the time to progression by nearly 3 months in patients with advanced HCC in 2008, no standard systemic chemotherapy in patients with advanced HCC had been established yet [20]. As Bruix et al. discussed, sorafenib is now considered the first-line treatment in patients with HCC who can no longer be treated with potentially more effective therapies across the world [21]. In our case, he received S-1 plus cisplatin as an adjuvant therapy after the second surgical procedures, and he additionally received sorafenib after the spreading of HCC metastasis to the bone and lung. In the same way, concerning the role of neoadjuvant chemotherapy in HCC patients, Samuel et al. concluded that there was no clear evidence to show that neoadjuvant therapy increases survival from HCC [22]. However, Williet et al. reported the first case of a patient with HCC with LN metastases treated by sorafenib combined with gemcitabine plus oxaliplatin, with a partial response, which allowed curative surgery [23], and it is still a matter of debate.

## Conclusions

In conclusion, our patient had an uncommon pattern of progression and growth of HCC, with LN metastases that comprised HCC and infiltrated the IVC and produced an IVC tumor thrombus. Surgeons should consider the possibility of LN skip metastases and infiltrative growth patterns with tumor thrombi after hepatectomies.

## Consent

Written informed consent was obtained from the patient for the publication of this report and any accompanying images.

## Abbreviations

AFP: α-fetoprotein; CT: Computed tomography; CTA: Computed tomography during arteriography; CTAP: Computed tomography during arterioportography; F-FDG: ^18^ F-fluorodeoxyglucose; HBV: Hepatitis B virus; HCC: Hepatocellular carcinoma; IVC: Inferior vena cava; LC: Liver cirrhosis; LNs: Lymph nodes; PET: Positron-emission tomography; PIVKA-II: Protein induced by vitamin K absence or antagonist II; PV: Portal venous; RFA: Radiofrequency ablation; RT: Radiotherapy; SBRT: Stereotactic body radiotherapy; TACE: Transcatheter arterial chemoembolization.

## Competing interests

The authors declare that they have no competing interests.

## Authors' contributions

SI and KI drafted the manuscript and made revisions. HO, HT, HA, TK, KI, KI, SI performed the surgery. HA and KC participated in the medical treatment. KA carried out the pathological examination. All authors read and approved the final manuscript.
